# Locally-Induced CaMKII Translocation Requires Nucleotide Binding

**DOI:** 10.3389/fnsyn.2020.00004

**Published:** 2020-02-07

**Authors:** Zachary T. Fitzgerald, Jacqueline K. Rose

**Affiliations:** Behavioral Neuroscience Program, Department of Psychology, Western Washington University, Bellingham, WA, United States

**Keywords:** CaMKII, ATP—adenosine triphosphate, glutamate, synaptic, catalytic activity, translocation, kinase activation

## Abstract

Calcium-calmodulin-dependent protein kinase (CaMKII) is a molecule involved in several cell processes including plasticity related to learning and memory. Activation of NMDA-type glutamate receptors results in translocation of CaMKII to synapses. However, there are at least two distinct mechanisms by which glutamate-dependent CaMKII translocation occurs: one well-studied process resulting from whole-cell glutamate stimulation and one resulting from brief, local glutamate application. Unlike the relatively fast CaMKII translocation seen following whole-cell glutamate delivery (seconds), local application results in CaMKII translocation that occurs gradually within 6–10 min. This locally-induced translocation of CaMKII requires L-type Ca^2+^ channel co-activation but does not rely on GluN2B receptor subunit expression, unlike translocation following whole-cell application of glutamate. The current study examined if nucleotide binding is necessary for locally-induced CaMKII translocation, similar to CaMKII translocation resulting from whole-cell glutamate application. Three different mechanisms of inhibition were employed: staurosporine (ATP inhibitor), CaMKII(281–302) peptide inhibitor and expression of the K42M mutation. Locally-induced CaMKII translocation was moderately suppressed in the presence of either the broad-spectrum kinase inhibitor staurosporine (100 nm) or the CaMKII(281–302) peptide inhibitor. However, expression of the catalytically dead K42M mutation that prevents ATP-binding to CaMKII, significantly inhibited locally-induced translocation. Thus, CaMKII translocation following brief, local glutamate application requires nucleotide binding, providing support for future research into the molecular mechanisms of this distinct form of CaMKII translocation.

## Introduction

Calcium-calmodulin-dependent protein kinase (CaMKII) is involved in several cellular processes and is abundantly expressed in neurons (Erondu and Kennedy, 1985). It is well-established that whole-neuron activation with glutamate or NMDA results in rapid translocation of CaMKII to synapses (within seconds) resulting from calcium influx through activated NMDA receptor channels (Shen and Meyer, 1999; Shen et al., 2000; Thalhammer et al., 2006). Whole-cell glutamate-induced CaMKII translocation requires nucleotide binding, Ca^2+^/CaM activation of the kinase and binding of CaMKII to NMDA receptor GluN2B subunits, a process that increases the ATP-binding affinity of CaMKII (Shen and Meyer, 1999; Shen et al., 2000; Bayer et al., 2001, 2006; Cheriyan et al., 2011; O’Leary et al., 2011; Halt et al., 2012). However, CaMKII translocation resulting from whole-cell activation does not require voltage-gated Ca^2+^ channel activation and further does not rely on CaMKII autophosphorylation at the T286 site (Shen and Meyer, 1999; Bayer et al., 2006; Thalhammer et al., 2006; Rose et al., 2009; O’Leary et al., 2011).

Weaker glutamate excitation protocols (e.g., single puff of glutamate *via* local pipette) can also activate CaMKII translocation that occurs on a much longer time scale (on the order of minutes; Zhang et al., 2008; Rose et al., 2009; She et al., 2012). Similar to whole-cell activation protocols, CaMKII translocation resulting from a single local puff of glutamate similarly requires Ca^2+^/CaM activation and calcium influx *via* NMDA receptor channels (Rose et al., 2009). However, unlike CaMKII translocation following whole-cell stimulation, locally-induced CaMKII translocation requires L-type calcium channel activation but not CaMKII-GluN2B binding (Rose et al., 2009; She et al., 2012) indicating that the molecular mechanisms of locally-induced synaptic CaMKII translocation differ somewhat from those involved in CaMKII translocation resulting from whole-cell activation.

Given the consistent finding that nucleotide binding is required for whole-cell glutamate-induced CaMKII translocation, it remains of interest to determine if locally-induced CaMKII translocation also requires nucleotide binding. To test this, a combination of approaches was performed. Local stimulation was delivered in the presence of either staurosporine (a competitive ATP inhibitor) or a peptide inhibitor (CaMKII281–302). As well, locally-induced CaMKII translocation trials were performed following expression of the K42M mutation with knockdown of endogenous CaMKII. Significant inhibition of CaMKII translocation resulted with K42M expression while only moderate inhibition was noted in the other conditions. The results from these trials contribute to uncovering the basic mechanisms of locally-induced synaptic CaMKII translocation.

## Materials and Methods

### Hippocampal Neuron Cultures

All experiments involving the use of rats and the procedures were approved by the Western Washington University Institutional Animal Care and Use Committee and were in strict accordance with the Guide for the Care and Use of Laboratory Animals described by the National Institutes of Health. E18 rat hippocampal neurons were isolated and cultured as described by Harms and Craig (2005) and Kaech and Banker (2006). Briefly, dissociated hippocampal neurons were plated onto poly-D-lysine-coated (MW >100,000; Sigma) glass coverslips at a density of 1.5 × 10^5^ cells per 60 mm dish in Neurobasal medium containing B27 (Gibco), 0.6 mM glutamine and 100 U ml^−1^/100 μg ml^−1^ penicillin/streptomycin. Neurons were maintained at 37°C and 5% CO_2_ and cultured with a glial feeder layer. One-third of the medium was exchanged weekly and 5 μM AP5 was added twice weekly. All experiments were performed on neurons 14–16 days *in vitro*. Neuron transfection occurred either ~72 h prior to experiments *via* Lipofectamine 3000 (Invitrogen) or by electronucleoporation just prior to plating using an Amaxa Nucleofector II (program O-005). Expression plasmids employed were either GFP-CaMKIIα (gift from A.M. Craig) or GFP-CaMKIIα with K42M mutation combined with shRNA for endogenous CaMKII knockdown (gift from K.U. Bayer; see Barcomb et al., 2014 for details).

### Drug Conditions

Glass micropipettes were shaped with a 3–5 μm opening similar to that used in Rose et al. (2009) using a micropipette puller (Model P-97, Sutter) just prior to imaging and loaded with glutamate plus glycine in ECS solution (10 μM glycine, 100 μM glutamate; ECS: 168 mM NaCl, 2.4 mM KCl, 10 mM HEPES, 10 mM D-glucose, 1.3 CaCl_2_, 1.3 MgCl_2_, pH 7.3). Drug conditions included 100 nM staurosporine (Sigma, CA, USA), a concentration previously reported to block CaMKII activity (Yanagihara et al., 1991) and peptide inhibitors CaMKII(290–309; sc3037, 1 μM, sequence: LKKFNARRKLKGAILTTMLA) and CaMKII(281–302; sc3039, 1 μM, sequence: MHRQEAVDCLKKFNARRKLKGA; Santa Cruz Biotechnology, Dallas, TX, USA) to inhibit calmodulin- or ATP-binding, respectively (Malinow et al., 1989). Staurosporine or peptide inhibitors were bath applied to neuron cultures at least 20 min prior to local glutamate stimulation and were included in the stimulation pipette. A locally-induced stimulation trial with an ECS vehicle control bath always preceded inhibitor/mutation trials.

### Imaging and Image Analysis

**Figure 1:**
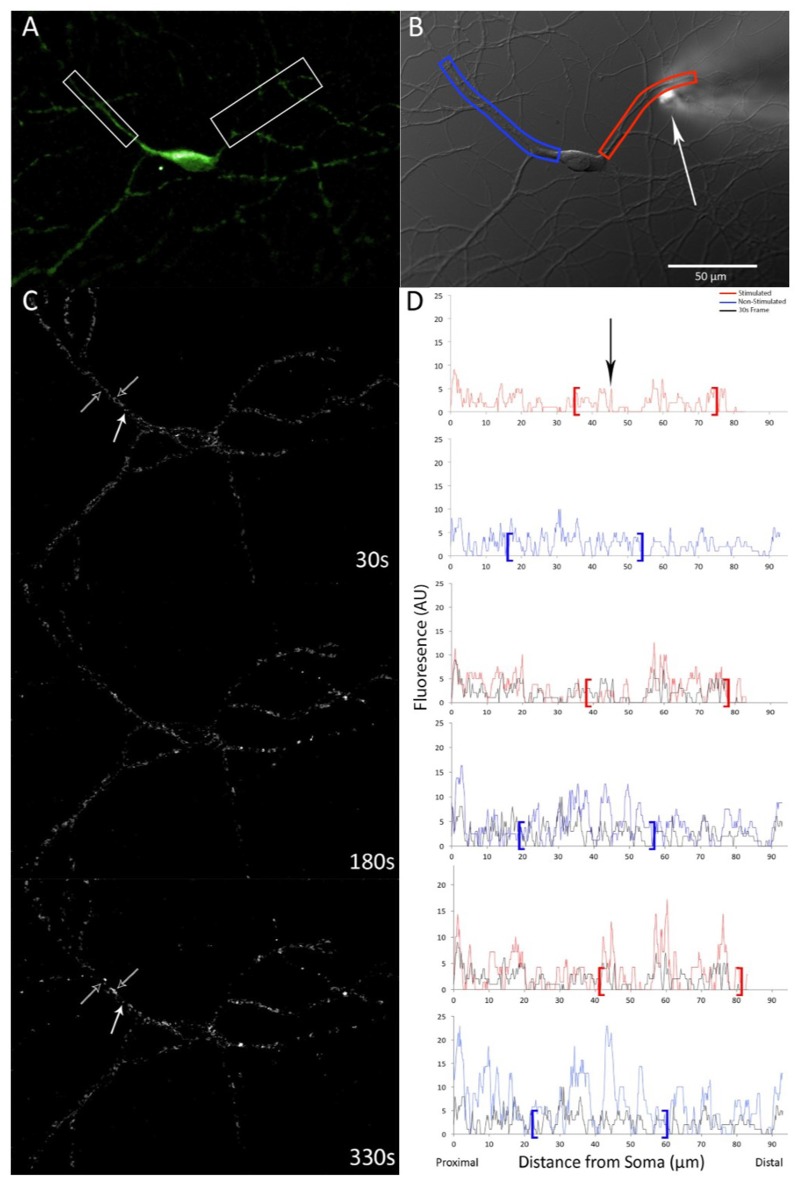
GFP-calcium-calmodulin-dependent protein kinase (CaMKII) expression and translocation after stimulation with 100 μM glutamate with 10 μM glycine *via* pipette. **(A)** GFP-CaMKII expression prior to stimulation. **(B)** DIC image of the same neuron with measured dendrites highlighted in red (right) and blue (left) and an arrow indicating the position of the micropipette. Scale bar = 50 μm. **(C)** Time-lapse subtraction images (current fluorescence level minus initial fluorescence level; increases in fluorescence appear whiter while decreases are darker/black). Hollow white arrows indicate dendrite locations that show increased relative fluorescence over time, solid white arrow indicates the location where relative fluorescence level decreases. **(D)** Linescan measures of the same neuron shown in **(C)** indicating fluorescence levels for respective dendrites, red (top) and blue (bottom), starting proximal to the cell body. Brackets denote regions that correspond to boxes shown in **(A,B)**. Black linescan trace indicates fluorescence levels measured at first post-stimulation image capture for comparison. Arrowhead indicates the location of the stimulation pipette.

Live-imaging of neurons was conducted on an Olympus IX-81 inverted microscope with epifluorescence. All images were collected with a Hamamatsu CCD camera using Metamorph software (version 7.6.5.0; Molecular Devices Inc., San Jose, CA, USA). Individual coverslips were mounted in an open chamber with a continuous circulating ECS bath. Once a single neuron was in focus, a DIC and a GFP fluorescence image were captured (Figure 1A). A micropipette connected to an air injector (microINJECTOR with MINJ-2 pulse-length control module; Tritech) was then lowered within ~5–10 μm of a dendrite (Figure 1B). Glutamate (plus glycine) solution was delivered *via* micropipette (100 ms at 100 psi). Within 1–2 s following stimulation, the micropipette was raised away from the neuron and time-series collection began with GFP images captured (500 ms exposure duration) once every 30 s (Figure 1C). Following the conclusion of the time series, an additional post-translocation DIC image was captured. Morphology filters were employed in Metamorph to produce a consistent measure of relative fluorescence over time. A correction function was employed in FIJI software (overall fluorescence level of each frame was compared to the first frame in a time series to determine a normalizing ratio; Schindelin et al., 2012) to account for any potential bleaching.

Images were analyzed using Metamorph software (version 7.6.5.0; Molecular Devices Inc., San Jose, CA, USA). Using the linescan function, a multipoint line with a width of 18 pixels was drawn along dendrites and fluorescence intensity was captured (Figure 1D). Linescan data processing was performed using a custom R script (see Supplementary Data Sheet 1) whereby fluorescence-expressing puncta (i.e., fluorescence peaks) were determined mathematically to avoid selection biases. Briefly, two moving averages for fluorescence intensity were computed along each dendrite; one a moving average of five pixels (approximate minimum size of fluorescence puncta; the first value was the average fluorescence intensity of the first five pixels, the next value being the average of the second to sixth-pixel intensity values, and so on) and the other a moving average of 31 pixels (fluorescence for short dendrite region). Each punctal fluorescence average (five pixels) was then divided by the corresponding 31-pixel average for that specific dendrite region with quotients greater than 1.1 being flagged as peaks (a point of fluorescence brightness as an indication of puncta). For confirmation that the mathematical determination reliably identified GFP-expressing puncta along dendrites, locations of flagged puncta were mapped and compared to the original fluorescence data.

Once puncta were identified, the fluorescence of puncta was calculated by dividing punctal fluorescence intensity by the total fluorescence for each frame (accounting for background and bleaching). An increase in punctal fluorescence intensity served as an indicator of CaMKII translocation and was calculated as the average relative peak fluorescence level for each dendrite normalized to initial peak fluorescence intensity. In the vehicle group, it was noted that GFP-CaMKII translocation was seen in all dendrites, both adjacent and non-adjacent to the stimulation site, with no significant difference in time of translocation initiation (*F*_(48,2)_ = 0.34, *p* > 0.10; Figures 1C,D). Thus, data were pooled across dendrites for each experimental condition.

The density of GFP-CaMKII puncta was calculated by dividing the number of identified puncta by the length of the dendrite measured using the MetaMorph linescan function yielding a value in puncta/μm. To compare relative GFP-CaMKII fluorescence intensity as well as GFP-CaMKII punctal density, a repeated measures mixed-design ANOVA was employed (with Greenhouse-Geisser correction) followed by tests of simple main effects with Šidák adjustment for multiple tests. According to Levene’s test, groups had equal variances over time. All statistics were performed using SPSS (version 25) and graphs were designed in Excel 2016.

#### Imaging and Image Analysis of T286A Expressing Neurons

For GFP-CaMKII expressing neurons co-transfected with T286A, live-cell imaging methods were as described above except imaging was performed on a Nikon TE300 microscope with a 40× 1.0 NA oil objective and Retiga EXi or Diagnostics Instruments SPOT cooled CCD camera using Metamorph software, as reported in Rose et al. (2009). As well, in these experiments, local pipette stimulation was delivered *via* a single pressure ejection of 10–20 psi and 10–25 ms *via* a General Valve Picospritzer as reported in Rose et al. (2009). Images were acquired at equal gain and contrast settings. Subtraction images (e.g., Figures 3C,D) were obtained by subtracting the pre-stimulus image from images captured at either an early (60 s) or late (360 s) time point. Images were analyzed for punctal fluorescence intensity and representative images were compiled for figures using FIJI.

**Figure 2:**
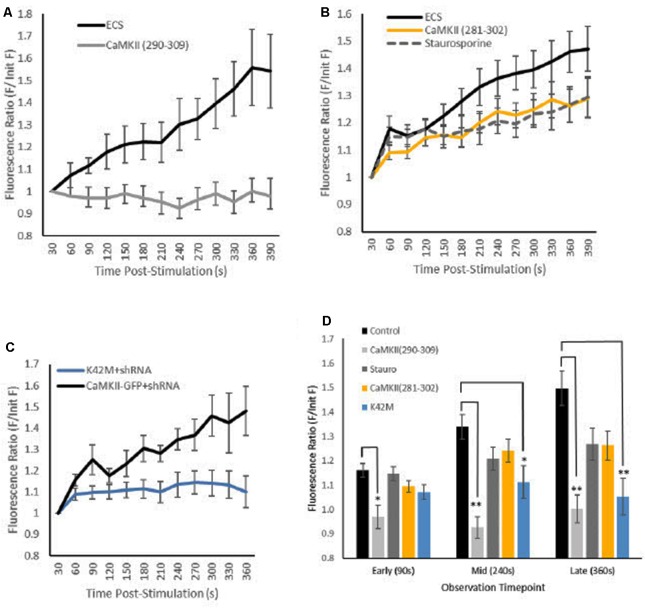
Effects of CaMKII inhibition on GFP-CaMKII translocation. Fluorescence levels **(A–C)** [Mean (SEM)] of GFP-CaMKII-expressing puncta relative to initial GFP-CaMKII fluorescence levels across post-stimulation time points captured at 30-s intervals. **(A)** GFP-CaMKII expression in control (ECS; *n* = 10) and in the presence of the CaMKII(290–309) peptide inhibitor (*n* = 10). **(B)** GFP-CaMKII in control (ECS, *n* = 14), staurosporine (*n* = 12) or in the presence of the CaMKII(281–302) peptide inhibitor (*n* = 10). **(C)** CaMKII knockdown and re-expression of GFP-CaMKII (*n* = 6) or GFP-CaMKII(K42M; *n* = 16). **(D)** Mean (±SEM) relative GFP-CaMKII punctal fluorescence levels at early (90 s), mid (240 s) and late (360 s) translocation time points by condition. ECS and GFP-CaMKII knockdown and re-expression conditions combined as there was no significant difference between these control conditions (*F*_(18,38)_ = 1.008, *p* > 0.40). *p < 0.05, **p < 0.01.

**Figure 3:**
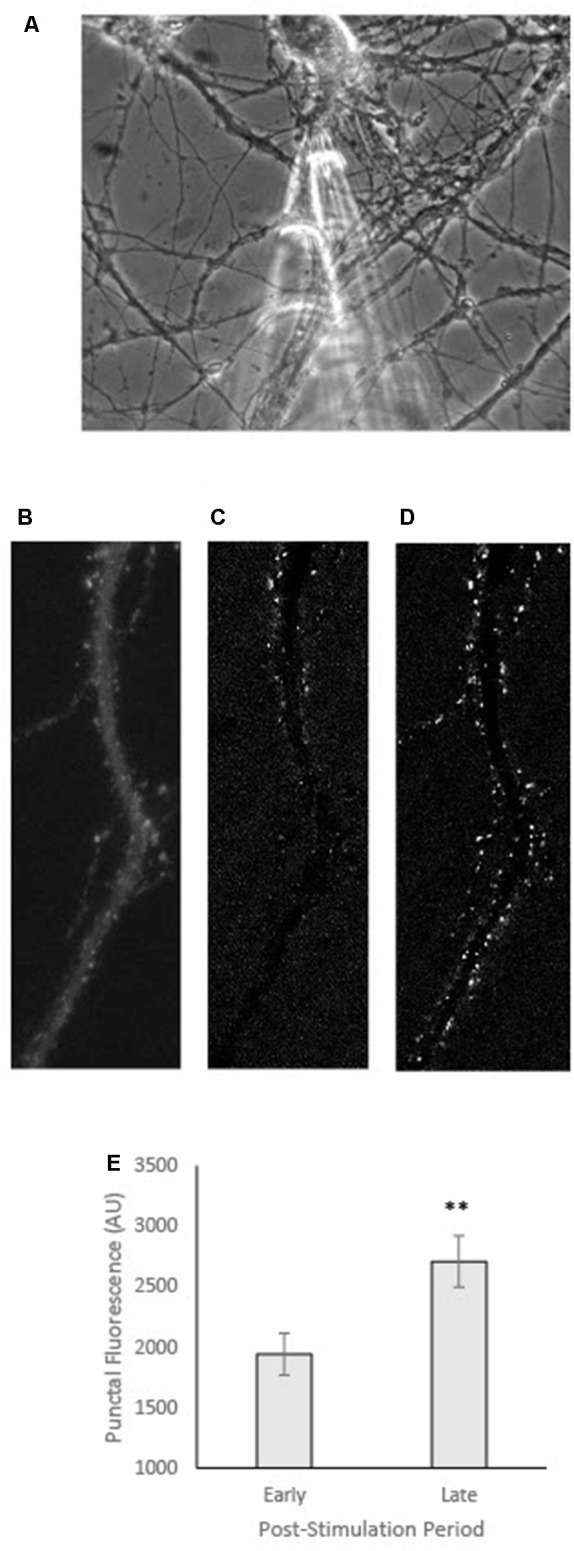
GFP-CaMKII translocation following local stimulation in neurons co-transfected with T286A. **(A)** Phase image of representative neuron showing micropipette captured prior to local stimulation. **(B)** GFP-CaMKII expression in the same neuron in **(A)** prior to stimulation. **(C)** Subtraction image (current fluorescence level minus initial fluorescence level; increases in fluorescence appear whiter while decreases are darker/black) captured within 60 s of local stimulation (early). **(D)** Subtraction image at 360 s post-stimulation (late). **(E)** Fluorescence intensity measured at an early (60 s) vs. late (360 s) time point following local stimulation. **p < 0.01.

## Results

CaMKII translocation was measured as an increase in fluorescence intensity of GFP-CaMKII expressing puncta following stimulation. Brief delivery of 100 μM glutamate plus 10 μM glycine *via* micropipette was sufficient to initiate a progressive translocation of GFP-CaMKII that reached maximum punctal fluorescence intensity within 5–6 min (Figures 1C,D). In dendritic regions where pre-stimulation GFP-CaMKII expression was diffuse, local glutamate plus glycine stimulation resulted in the gradual appearance of GFP-CaMKII expressing puncta while previously bright puncta frequently showed an increase in fluorescence intensity, although occasional decreases in fluorescence at these early-observable puncta were also seen (Figure 1C). Although an increase in puncta number was anticipated, the density of GFP-CaMKII expressing puncta did not show a significant difference between groups (*F*_(4,69)_ = 0.878, *p* > 0.40, with Greenhouse–Geisser sphericity correction).

The presence of inhibitors produced differential effects on locally-induced CaMKII translocation resulting in a significant interaction between experimental conditions across time (*F*_(12.25,211.24)_ = 3.31, *p* < 0.001, with Greenhouse–Geisser sphericity correction; Figures 2A–C). Simple main effects tests confirmed that inhibition of calmodulin blocked CaMKII translocation; the CaMKII(290–309) peptide inhibitor was sufficient to block translocation measured at early (90 s), mid (240 s) and late (360 s) time points (Figure 2D). Specifically inhibiting nucleotide binding to CaMKII with the K42M mutation did block locally-induced CaMKII translocation but only at the mid- and late time points (Figure 2D). The staurosporine and the CaMKII(281–302) peptide inhibitor conditions produced only moderate CaMKII translocation that was not statistically significant from translocation seen in the vehicle condition or the inhibited translocation in the calmodulin peptide inhibitor condition (*p* > 0.20). These results demonstrate that both CaMKII activation and nucleotide-binding are necessary for locally-induced CaMKII translocation.

## Discussion

The results confirm that a single brief (100 ms) application of 100 μM glutamate plus 10 μM glycine *via* local pipette is sufficient to trigger gradual CaMKII translocation by 6 min post-stimulation (Figure 2A). As expected, inhibition of calmodulin with the CaMKII(290–309) peptide inhibitor effectively prevented locally-induced CaMKII translocation (Figure 2A) similar to what has been previously reported with another calmodulin activation inhibitor of CaMKII, KN93 (Rose et al., 2009).

Overall, results from ATP inhibitor conditions were mixed; however, notably the K42M mutation that prevents nucleotide binding to CaMKII inhibited locally-induced CaMKII translocation (Figure 2C). This result was predicted as previous reports have shown that K42M prevents CaMKII translocation or aggregation following whole-cell stimulation with glutamate (Hudmon et al., 2005; Vest et al., 2009; O’Leary et al., 2011). However, in the current study inhibition of CaMKII translocation was not immediately notable as the K42M condition only reached significance compared to the vehicle condition after ~4 min post-stimulation (Figure 2D). Given the small increase in CaMKII punctal expression seen in the vehicle condition at the early time point (90 s), it is difficult to ascertain if the lack of a significant difference from the K42M condition at the early time point is meaningful. However, others have reported that although K42M prevents ATP-binding and kinase activation, it does not prevent association with F-actin or microtubules (O’Leary et al., 2011; Lemieux et al., 2012; Khan et al., 2016). Thus, it is possible that the lack of significant difference at the early time point for the K42M condition may be due to some weak initial ATP-independent peri-synaptic translocation of CaMKII.

Unlike the K42M condition, the staurosporine and the CaMKII(281–302) peptide inhibitor conditions resulted in moderate CaMKII translocation levels that were not significantly different from either vehicle or the CaMKII(290–309) calmodulin inhibitor condition (Figures 2B,D). There are several possible explanations for this outcome, including the possible role(s) of other kinases. In the current study, both staurosporine and the CaMKII(281–302) peptide inhibitor were employed at concentrations previously reported to be effective to inhibit CaMKII activation and CaMKII-ATP binding (Yanagihara et al., 1991; Smith et al., 1992; Barcomb et al., 2013). Given that ATP inhibition in these conditions is non-specific, perhaps some negative regulator of locally-induced CaMKII translocation was also inhibited. For example, phosphorylation at CaMKII site T305/T306 (α and β subunits, respectively) inhibits activation of CaMKII by calmodulin, predisposes neurons to long-term depression and decreases synaptic localization of CaMKII when co-expressed with the constitutively active T286D mutation (Patton et al., 1990; Hanson et al., 1994; Pi et al., 2010; Barcomb et al., 2014). As well, CaMKII translocation to microtubules resulting from whole-cell KCl stimulation is inhibited by the constitutively active T305D mutation (Lemieux et al., 2012). Thus, it is possible that the moderate decrease in locally-induced CaMKII translocation noted in the present study is the result of some ATP-dependent process that normally inhibits CaMKII translocation.

In addition to ATP inhibition, both staurosporine and the CaMKII(281–302) peptide inhibitor block the catalytic activity of the CaMKII molecule (Malinow et al., 1989; Barcomb et al., 2013). Thus, it is possible that the moderate effects of these inhibitors reflect some requirement of autophosphorylation for locally-induced translocation. However, co-expression of T286A (Figures 3A–B), a modification that impairs autophosphorylation (Giese et al., 1998), has little to no effect on locally-induced translocation (Figures 3C–E) similar to what has been previously reported with T286A for CaMKII translocation following global bath stimulation (Shen and Meyer, 1999).

Staurosporine alone has previously been reported to result in CaMKII translocation and potentially facilitates CaMKII binding to GluN2B receptor subunits (Barcomb et al., 2013). For the current study, it is possible that staurosporine itself somehow primed neurons for CaMKII translocation while inhibiting any ATP-dependent mechanisms. It is interesting that in the current study, bathing neurons for several minutes in staurosporine resulted in a moderate level of CaMKII translocation that developed gradually and appeared to be initiated by brief, local glutamate application. This is different to the seemingly maximal CaMKII translocation produced by staurosporine alone as reported in Barcomb et al. (2013); however, this difference may be a reflection of staurosporine concentrations as the current study used a staurosporine concentration of 100 nM while Barcomb et al. (2013) reported CaMKII translocation comparable to whole-cell glutamate-induced levels following bath delivery of 2 μM staurosporine. Taken together, the variability of results noted here is consistent with the equivocal outcomes others have reported with ATP inhibitors in whole-cell glutamate stimulation trials providing evidence that mode of action for inhibitors similarly must be considered for locally-induced CaMKII translocation.

Results from this study confirmed that ATP binding is necessary for locally-induced CaMKII translocation. Additional research is needed to identify phosphorylation sites involved. One possibility is autophosphorylation of the T253 site as this develops over a similar time scale of minutes (Migues et al., 2006). An alternative possibility is that local stimulation affects some part of the basal exchange of CaMKII between dendritic spines and shafts (a process that normally occurs between 1–5 min; Sharma et al., 2006; Lee et al., 2009; see Hell, 2014), thus resulting in a gradual accumulation of the molecule at spines. In addition to phosphorylation sites, the synaptic binding partner(s) of CaMKII following locally-induced translocation remains unknown. Studies into mechanisms of neuroplasticity have identified the K42 site as important for long-term potentiation and correlated spine enlargement (Yamagata et al., 2009; Kabakov and Lisman, 2015). Thus, it is of interest to identify mechanisms affected by brief, local glutamate stimulation and discern the functional purpose of this form of CaMKII translocation.

## Data Availability Statement

The datasets generated for this study are available on request to the corresponding author.

## Ethics Statement

The animal study was reviewed and approved by Western Washington University Institutional Animal Care and Use Committee in strict accordance with the Guide for the Care and Use of Laboratory Animals described by the National Institutes of Health.

## Author Contributions

ZF performed all experiments, prepared dissociated hippocampal neuron cultures and designed manuscript figures under the direction and guidance of JR. Image processing and data extraction performed by ZF and JR. Initial and ongoing instruction and direction of experimental procedures, experiment conceptualization, statistical analyses and manuscript text composition performed by JR.

## Conflict of Interest

The authors declare that the research was conducted in the absence of any commercial or financial relationships that could be construed as a potential conflict of interest.
