# Supplementary material for: Locally-Induced CaMKII Translocation Requires Nucleotide Binding
Source: Front Synaptic Neurosci. 2020 Feb 7;12:4. doi: 10.3389/fnsyn.2020.00004 (PMC7019030; doi:10.3389/fnsyn.2020.00004)
Supplement: Supplementary file 1 [file Data_Sheet_1.PDF]

```
##########################Import Raw Data##################
#Read data in
data_raw <- as.matrix(read.csv("1.csv",F))

# Find separation line:
id_sep <- which(data_raw[,2] == "")
id_sep <- c(id_sep, nrow(data_raw)+1)

# Break that data down
data_list <- as.list(1:(length(id_sep)-1))
for (i in 1:(length(id_sep)-1)) {
   data_list[[i]] <- data_raw[(id_sep[i]+2):(id_sep[i+1]-1), c(1:3)]
   data_list[[i]] <- apply(data_list[[i]], 2, as.numeric)
}

#Add One to Remove Zeros
for (i in 1:(length(id_sep)-1)) {
   data_list[[i]][,3] <- data_list[[i]][,3]+1
}

#Bleaching Correction
if(file.exists("bc.csv")){
   bleach_correction <- unname(unlist(c(1, read.csv("bc.csv",F))))
   for(i in 1:length(bleach_correction)){
      data_list[[i]][,3] <- data_list[[i]][,3]*bleach_correction[i]
   }
}



##########################Find Percentile##################
percentile <- function(x, k) {
   a <- sort(x)
   b <- k*length(x)
   a[b]
}

##########################Moving Average##################
mAvg <- function(x, w) {
   as.vector(c(rep(NA, trunc((w-1)/2)),rollapply(x, w, mean), rep(NA, trunc((w-1)/2))))
}

##########################Find Peaks####################
findPeaks <- function(t) {
   #By mAvg
   x <- 1:length(data_list[[t]][,1])
```

```
  y <- data_list[[t]][,3]
  top_mAvg <- mAvg(y,5)
  bot_mAvg <- mAvg(y,31)

  # Make Matrix of Peak Locations
  peaks_raw <- as.matrix(cbind(row(as.matrix(y)), y[ifelse(1.1>top_mAvg/bot_mAvg,
row(as.matrix(y)), NA)]))
  peaks_chng <- cumsum(rle(is.na(peaks_raw[,2]))$lengths)

  #Convert Peaks to List
  peaks_list <- as.list(seq(1, length(peaks_chng)/2))
  temp <- list(0)

  for(i in seq(1, length(peaks_chng)-1, 2)) {
    peaks_list[[ceiling(i/2)]] <- peaks_raw[(peaks_chng[i]+1):peaks_chng[i+1],]
    ifelse(length(peaks_list[[ceiling(i/2)]]) < 5, temp <- c(temp,ceiling(i/2)), 1)
  }

  temp[1] <- NULL
  peaks_list[c(unlist(temp))] <- NULL

  #Use Only Max Value as Peak
  peaks_point <-as.list(1:length(peaks_list))

  for(i in   1:length(peaks_list)) {
    peaks_point[[i]] <- peaks_list[[i]][which.max(peaks_list[[i]][,2]),]
  }
  peaks <- matrix(unlist(peaks_point), c(length(peaks_point), 2), byrow=T)
  colnames(peaks) <- c("Distance", "Fluorescence")
  peaks
}

###########################Relative Fluorescence####################
relFluorescence <- function(t) {
  #Definitions
  peaks <- findPeaks(t)
  s_peaks <- 1:length(peaks[,1])
  e_peaks <- 1:length(peaks[,1])

  #Average Fluorescence Data Around Peaks
  for(i in 1:length(peaks[,1])) {
    s_peaks[i] <- mean(data_list[[1]][(peaks[i,1]-2):(peaks[i,1]+2),3])
    e_peaks[i] <- mean(data_list[[t]][(peaks[i,1]-2):(peaks[i,1]+2),3])
  }

  rel_fluor <- e_peaks/s_peaks
```

```
    rel_fluor <- cbind(peaks[,1],rel_fluor)
    colnames(rel_fluor) <- c("Distance", "Relative Fluorescence")
    rel_fluor
}


##########################Average Relative
Fluorescence####################
avgPeakFluorescence <- function() {
    avgRelFluor <- 1:length(data_list)
    for(i in 1:length(data_list)) {
        avgRelFluor[i] <- mean(relFluorescence(i)[,2])
    }
    avgRelFluor <- cbind(1:length(data_list),avgRelFluor)
    colnames(avgRelFluor) <- c("Frame", "Average Fluorescence")
    avgRelFluor
}
##########################Individual Relative
Fluorescence####################
indRelFluor <- function() {
    #Definitions
    avgRelFluor <- avgPeakFluorescence()
    max <- which.max(avgRelFluor[,2])
    peaks <- findPeaks(max)
    ipeak <- 1:length(data_list)
    peak_max <- 1:length(peaks[,1])

    #Find individual peaks' brightest time
    for(i in 1:length(peaks[,1])) {
        for(j in 1:length(data_list)) {
            ipeak[j] <- data_list[[j]][peaks[i,1],3]
        }
        peak_max[i] <- which.max(ipeak)
    }

    indRelFluor <- 1:length(peaks[,1])
    for(i in 1:length(peaks[,1])) {
        indRelFluor[i] <- mean(data_list[[peak_max[i]]][(peaks[i,1]-2):(peaks[i,1]+2),3])/
            mean(data_list[[1]][(peaks[i,1]-2):(peaks[i,1]+2),3])
    }

    indRelFluor <- cbind(peaks[,1],indRelFluor)
    colnames(indRelFluor) <- c("Distance", "Relative Fluorescence")
    indRelFluor
}
###########Translocation Times##################
```

```
tTimes <- function() {
   #Definitions
   avgPeakFluor <- avgPeakFluorescence()[,2]
   end <- which.max(avgPeakFluor)
   start_point <- .1*(avgPeakFluor[end]-avgPeakFluor[1])+avgPeakFluor[1]
   change <- cumsum(rle(avgPeakFluor > start_point)$lengths)
   start <- change[1]+1
   times <- c(start,end)
   names(times) <- c("Start","End")
   return(times)
}

###################Density#################
pDensity <- function() {
   #avgPeakFluor <- avgPeakFluorescence()[,2]
   #end <- which.max(avgPeakFluor)
   #d <- length(end)/length(data_list[[1]][,1])

   d <- NULL
   for(i in 1:length(data_list)){
      d <- c(d, length(findPeaks(i)[,1])/length(data_list[[1]][,1]))
   }

   d <- as.matrix(d)
   colnames(d) <- "Peaks/Pixel"
   return(d)
}
```
